# Low-Grade Pseudomyxoma Peritonei Behaving as a High-Grade Disease: A Case Series and Literature Review

**DOI:** 10.3390/curroncol30110726

**Published:** 2023-11-18

**Authors:** Petros Bangeas, Dimitrios Kyziridis, Apostolos Kalakonas, Apostolis A. Tentes

**Affiliations:** 1 University Surgery Department, Papageorgiou Hospital, Aristotle University of Thessaloniki, 54453 Thessaloniki, Greece; 2 Department of Surgical Oncology, Peritoneal Surface Malignancy Program, Euromedica Kyanos Stavros, 54454 Thessaloniki, Greece; dkyziridis@gmail.com (D.K.); tolistentes@gmail.com (A.A.T.); 3 Department of Anaesthesiology, Euromedica Kyanos Stavros, 54454 Thessaloniki, Greece; akalakonas@gmail.com

**Keywords:** LAMN, cytoreductive surgery, HIPEC, pseudomyxoma peritonei

## Abstract

Patients with low-grade appendiceal mucinous carcinomas (LAMNs) treated with cytoreductive surgery (CRS) and hyperthermic intraperitoneal chemotherapy (HIPEC) have a favorable prognosis. However, a subgroup of patients presents a clinically aggressive course with disease progression despite receiving treatment. The purpose of this study is to report the experience of clinically aggressive LAMN patients treated by the same team, and to present a review of the literature. The cases of four patients with clinically aggressive LAMNs were reviewed. Clinical and histopathological characteristics were re-examined. Recurrences and the time of recurrence, as well as the survival time, were recorded. These patients were four men with clinically aggressive LAMNs treated with CRS plus HIPEC. One of them underwent CC-0 surgery, two underwent CC-1 surgery, and one underwent CC-3 surgery. All patients received systemic chemotherapy after surgery. Recurrence was recorded in three of the patients within 4–23 months after the initial treatment. Two of the patients underwent secondary CRS. Three patients died of disease recurrence within 13–23 months, and one is alive with a disease relapse at 49 months after his initial surgery. LAMNs were identified in both the initial specimens and the specimens obtained during reoperation. The prognosis of LAMN patients treated with CRS plus HIPEC is favorable. A small number of patients present a clinically aggressive course that is unresponsive to any treatment. Molecular and genetic studies are required to identify this group of LAMN patients who have an unfavorable prognosis.

## 1. Introduction

The peritoneum is the largest membrane in the human body. In men, it is a closed space; in women, there is a connection between the peritoneal space and the external genitalia. The peritoneum is divided into parietal and visceral layers. For peritoneal tumors, a distinction is made between primary and secondary tumors [1,2,3,4]. 

Pseudomyxoma peritonei (PMP) is a very rare secondary tumor. Limited cases of primary PMP without distal metastases have also occurred. Its diagnosis is not based on pathological features, but instead on clinical features defined by a mucinous appearance, usually leading to abdominal distension and bowel obstruction. In the vast majority of patients, a mucinous tumor originates from the appendix, while in a few cases, it originates from the ovaries, the pancreas, the gallbladder, the bowel, or an unknown site. A mucinous tumor is not always obvious because of the large volume of the tumor. Pseudomyxoma peritonei syndrome is a clinical entity originating from an appendiceal mucinous tumor [1]. It is characterized by a redistribution phenomenon in which there is the accumulation of large-volume mucinous tumors at the greater omentum, the undersurface of the hemidiaphragm, the pelvis, and the left paracolic gutter, along with an absence of tumors from sites with intense motility, such as the small bowel [2].

Most patients are asymptomatic, while a small proportion present symptoms of appendicitis. Werth was the first to use the term pseudomyxoma in 1884 to describe a case of ovarian neoplasm. In 1937, Robert Michaelis Von Olshausen proposed a possible hypothesis of the pathophysiology of PMP and fully described this disease. Today, the classic theory about the distribution of PMP is the redistribution phenomenon theory. This phenomenon results from free-floating epithelial cells’ movement into the peritoneal fluid gravity, progressively leading to the “Jelly-Belly” condition. According to the redistribution phenomenon theory, organs and surfaces in the peritoneal cavity could be involved with tumor cells [1,2,3]. 

The pathology of PMP was reported by Ronnet et al. in 1995, who classified PMP into three entities: disseminated peritoneal adenomucinosis (DPAM), peritoneal mucinous carcinomatosis (PMCA), and an intermediate hybrid morphological type (PMCA-I/D) [4,5,6,7].

This classification is based on the histology of the peritoneal disease present in patients rather than the primary tumor, which is unusual in oncology [4]. Various medical societies have attempted to classify PMP according to current oncologic requirements. The current classification specifies that the peritoneal disease and the appendiceal tumor should be reported separately [4,6]. Based on the modified Carr classification [4], peritoneal disease is categorized as follows: (1) acellular mucin, (2) low-grade mucinous carcinoma peritonei (LAMN), (3) high-grade mucinous carcinoma peritonei (HMAC) or peritoneal mucinous carcinomatosis (PMCA), and (4) high-grade mucinous carcinoma peritonei with signet-ring cells (PMCA-S). LAMN neoplasms display benign features, while the term PMP should be avoided with acellular mucin unless the syndrome is clinically obvious [5]. All the above histopathological categories have a profound impact on survival, provided that patients are treated with CRS and HIPEC [7]. 

CEA and CA 19-9 were evaluated and have been found to have a positive correlation with PCI score and overall survival in most reports [8,9,10,11,12,13].

Due to the rarity of PMP, consensus has a significant impact on the management of the disease. Currently, there are five up-to-date consensuses around the world, which were published by the PSOGI, CCWG, CACA, LARPD, and BSSO. Since the first consensus published by the PSOGI, a combination of cytoreductive surgery procedures (CRSs) and hyperthermic intraperitoneal chemotherapy (HIPEC) provides a treatment option that is regarded as the standard of care. However, the role of HIPEC remains controversial. 

In 2004, Mohamed et al. studied 11 cases of disseminated peritoneal adenomucinosis (DPAM) that succumbed to a rapidly progressive disease [9]. The purpose of our study is to report four patients with LAMNs who were treated with extensive cytoreduction and HIPEC and found to have clinically aggressive PMP. This study highlights the significance of recognizing and addressing this particular PMP subtype early on. 

## 2. Materials and Methods

### 2.1. Clinical Features

Our study group consisted of 4 patients with a pathological diagnosis of DPAM, who were selected out of 41 patients (9.76%) with LAMNs.

Despite the initial cytoreduction combined with HIPEC and intravenous chemotherapy, these four patients experienced recurrence of invasive disease. These patients were matched for age, gender, and co-morbid factors (smoking history, alcohol consumption, diabetes, and cardiovascular disease). Co-morbid factors (smoking history, alcohol consumption, diabetes, and cardiovascular disease) were recorded in detail, and then, the patients were assessed according to the ASA classification. Prior surgical score (PSS), peritoneal cancer index (PCI), and completeness of cytoreduction score (CC) were validated.

Many cytoreductions were recorded. In patients with PSS-0, the diagnosis of carcinomatosis was based on biopsies, while in patients with PSS-1, it was based on previous laparotomy without resections. PSS-2 indicated laparotomy with limited resections, and PSS-3 consisted of patients with full cytoreduction procedures (more than 5 regions) [10].

### 2.2. Cytoreduction Protocol

All patients were treated according to the standard protocol for cytoreductive surgery and HIPEC. Complete CRS may require six steps of peritonectomy, and the target is to eliminate all tumor deposits in the peritoneal cavity. Peritonectomy procedures may involve greater omentectomy with splenectomy, left and/or right upper quadrant peritonectomy, lesser omentectomy with cholecystectomy, pelvic peritonectomy with rectosigmoid or subtotal colectomy, and total or partial resection of the stomach. The same surgical team performed these procedures. 

Every patient was provided with thromboprophylaxis and perioperative antibiotics. The cytoreductive procedure was performed with the patient in the lithotomy position, and a midline incision was made that extended from the xiphoid to the pubis. After surgical lysis of the adhesions, the extent of the peritoneal disease was recorded according to the PCI. The tumor volume was assessed as small or large volume. Tumors with a maximal diameter less than 0.5 cm were classified as small-volume tumors, while those with a maximal diameter greater than 0.5 cm or confluence tumor masses of any diameter were classified as large-volume tumors. The resection of peritoneal disease was possible using the standard peritonectomy procedures [11]. After surgical resection of the tumor, the completeness of cytoreduction was assessed using the CC-score. CC-0 surgery indicated patients without macroscopically visible residual tumors. CC-1 surgery indicated patients with residual tumors that had a maximal diameter < 0.25 cm. CC-2 indicated residual tumors > 0.25 cm but < 2.5 cm, while CC-3 indicated residual tumors > 2.5 cm [10,12]. After tumor resection, HIPEC was performed for 90 min at 42.5–43 °C. HIPEC was administered using the open abdominal (Coliseum) technique. A heat circulator with two roller pumps, one heat exchanger, one reservoir, an extracorporeal system with two inflow and two outflow tubes, and 4 thermal probes was used for HIPEC (Sun Chip, Gamida Tech, France). A prime solution consisting of 2–3 L of normal saline or Ringer’s lactate solution was instilled prior to the administration of the cytostatic drug, and as soon as the mean abdominal temperature reached 40 °C, the cytostatic drugs were instilled in the abdomen. Mit-C (15 mg/m^2^) and doxorubicin (15 mg/m^2^) were used in HIPEC, and 5-FU (400 mg/m^2^) plus leucovorin (20 mg/m^2^) were given intravenously. Bi-cavitary HIPEC was performed in those cases where the diaphragm was opened during the subdiaphragmatic peritonectomy procedure. The reconstruction of the continuity of the gastrointestinal tract was performed after the completion of HIPEC. Proximal stoma was always performed if more than two anastomoses needed to be protected. All patients remained in the ICU for at least 24 h until hemodynamic stabilization. The morbidity and in-hospital mortality rates were carefully recorded. Patients with CC-2 or CC-3 surgery were treated with systemic chemotherapy after the initial treatment.

All patients were followed up every 3–4 months in the first year after the initial treatment, and every 6 months afterward. The follow-up included physical examination; thoracic and abdominal CT, MRI, or PET-CT scan; hematologic and biochemical examinations; and analysis of tumor markers (CEA, CA 19-9, and CA-125). Recurrences and the sites of recurrence were recorded in detail.

All specimens were examined in detail. The study recorded vital information concerning the tumor, including its subtype, degree of differentiation, the number of resected tumors, infiltrated lymph nodes, as well as the site and depth of tumor infiltration. The findings not only provide a comprehensive understanding of the tumor’s characteristics but also serve as a crucial reference point for future studies.

All patients signed an informed consent, and the Hospital’s Ethical Committee approved the study.

### 2.3. Statistical Analysis

Means with standard deviation or medians with interquartile range were reported for continuous variables, while frequencies with percentages were used for categorical variables in the descriptive statistics of patient demographics and disease characteristics. Statistical analysis was performed using SPSS version 25 (SPSS Inc., Chicago, IL, USA).

## 3. Results

Our study group consisted of four male patients (study group, 9.75%), who were selected from 41 LAMN patients (37 patients; control group, 90.24%) treated by our team from 2005 to 2018 (Table 1). The mean age at diagnosis was 48.5 years (range of 40–63 years). 

In our study group, no patient had a history of smoking or alcohol consumption, but one patient had intermittent atrial fibrillation. Two patients were treated with neo-adjuvant chemotherapy with oxaliplatin, 5-FU, and leucovorin, which offered significant benefits by reducing the extent and volume of the tumor, as shown on CT scans. The average PSS score was 0.5, the mean PCI score was 30 (SD 5.39), and the mean cytoreduction procedure duration was 8.2 h. Upon initial diagnosis, two patients were assessed as PSS-0, while the other two patients were assessed as PSS-1. However, two patients who underwent reoperation were later assessed as PSS-3. 

One patient underwent CC-0 surgery, which included a bilateral subdiaphragmatic peritonectomy procedure, a greater omentectomy with splenectomy, a lesser omentectomy, a cholecystectomy and resection of the omental bursa, a bilateral lateral peritonectomy, a right colectomy, a pelvic peritonectomy, and a total gastrectomy with loop ileostomy that was reconstructed four weeks later. 

Two patients underwent CC-1 surgery because we assumed that, without this surgery, a small-volume residual tumor would be left behind very close to the mesenteric edge of the small bowel, although no visible tumor was identified after resection. Both patients underwent a bilateral subdiaphragmatic peritonectomy procedure, a greater omentectomy plus splenectomy, a cholecystectomy with omental bursectomy, a bilateral lateral peritonectomy procedure, a pelvic peritonectomy, and a subtotal colectomy. One of them underwent an additional total gastrectomy with loop ileostomy that was reconstructed five weeks later. The other patient underwent additional segmental intestinal resection. All the above patients received HIPEC. The last patient underwent CC-3 surgery without HIPEC because he had a large-volume tumor in and around the hepatoduodenal ligament and which was strictly adherent to the inferior vena cava, which made a potentially curative resection impossible. This patient was offered a palliative subtotal colectomy, a greater omentectomy with splenectomy, and an ileostomy. The mean duration of all surgical operations was 8.2 h. Histopathologically, all the resected specimens were infiltrated by mucin. In two specimens, the infiltration of the peritoneal surfaces of the small and large bowels and the mesentery was visible. In one of the specimens, four infiltrated lymph nodes were retrieved, while in the other three, the resected lymph nodes were normal. The mean total number of resected lymph nodes was 74.75 (35–159). Two patients experienced complications of urinary infection.

Two patients were re-operated on because of recurrence. In one of the patients, who had undergone CC-0 surgery, recurrence was recorded at 23 months. This patient underwent CC-0 surgery plus HIPEC in which the left rectus abdominal muscle was resected due to a tumor originating from its upper part and entirely invading the left part of the muscle. This patient relapsed within six months after the second cytoreduction. The other patient, who had undergone CC-1 surgery, presented with recurrence after 6 months. This patient underwent a segmental intestinal resection, which was assessed as a CC-3 surgery, and died 23 months after the initial surgery. All resected specimens were LAMNs. Currently, one patient is still alive, with disease recurrence 49 months after his initial surgery. The other three patients died within 13, 19, and 23 months of their initial surgery. Both patients who had undergone CC-1 surgery presented multiple segmental intestinal obstructions, which were not amenable to surgical management. The follow-up period spanned 50 months, with a median survival rate of 26.75 months (SD 16.26) and a 5-year survival rate of 25%. Table 2 presents a comprehensive breakdown of the study group with clinically aggressive PSM and the control group with other LAMN cases analyzed in our study.

## 4. Discussion

Classification of PMP has been a topic of controversy for many years. The most widely accepted classification of PMP is presented in Table 3 [13,14,15].

Cytoreductive surgery and perioperative intraperitoneal chemotherapy are considered the standard of care for PMP. The histological characteristics of tumors are crucial in deriving an appropriate treatment strategy, with invasive histological types requiring more aggressive surgical interventions. 

In the past, PMP was thought to be a benign disease that could be treated by means of debulking and the evacuation of mucinous ascites [13,14,15]. However, this disease progresses rapidly, requiring aggressive debulking surgical operations followed by various adjuvant treatments, which achieves prolonged survival in 20–30% of cases [13,14]. The exact reason for the aggressive behavior of this tumor has not been thoroughly studied [9,10,11,12,13,14,15].

Mesothelial cells are responsible for synthesizing extracellular matrix molecules, which form a protective and non-adhesive surface for internal organs and the surrounding tissues. However, the peritoneum can be exposed to various stressors and aggressors, leading to the activation of mesothelial cells. Chronic inflammation and scarring could occur if the immune response fails to eliminate them. Activated cells promote cell adhesion, invasion, and proliferation and can promote metastasis. During surgical procedures, there exists a potential risk of tumor perforation and the transection of lymphatic vessels, which can inadvertently result in the escape of cancer cells into the abdominal cavity. This unintended release of cancer cells can cause grave concerns as it can pave the way for the spread of cancer to other parts of the body, leading to metastatic disease [14,15,16].

The scientific literature has discussed the assessment of the biological behavior of LAMNs, indicating that, in most cases, this neoplasm presents benign features. However, it is essential to note that ruptures or perforations of LAMNs can lead to intraperitoneal dissemination of mucin [10,11,12,13,14,15,16,17].

The aggressive behavior of these tumors appears to be influenced by several factors, namely the discharge of mucus and the surgical manipulations performed during an intervention. These factors activate mesothelial cells, thus contributing to tumor aggressiveness. It is critical to consider these factors when assessing the risk of aggressive behavior in tumors [16].

Patients with PMP may remain asymptomatic for many years, but this disease almost always recurs. Many patients ultimately die of intestinal obstruction. Repeated debulking operations become ineffective because the disease recurs, usually more aggressively. The lysis of the adhesions is usually impossible, or it results in bowel injury and subsequent fistula formation [16,17]. Cytoreductive surgery in combination with perioperative intraperitoneal chemotherapy has been established as the standard treatment for PMP. The addition of early postoperative intraperitoneal chemotherapy (EPIC) has been shown to provide additional survival benefits to patients with LAMNs [17,18]. In PMP, survival depends mainly on the tumor grade. The majority of long-term survivors are those with LAMNs [3,7]. According to the Ronnett classification, low-grade tumor cells do not have adhesion molecules on their surface, in contrast to high-grade tumor cells. As a consequence, low-grade cancer emboli cannot seed on peritoneal surfaces with intense motility. On the contrary, high-grade tumor cells are usually found on peritoneal surfaces, including those with intense motility, such as the small bowel [18,19]. The current PMP classification is different from the Ronnett classification (Table 1) for mucinous tumors, although LAMN has histopathological resemblance to DPAM [3,4,5]. DPAM cancer emboli are never found adherent to peritoneal surfaces with intense motility in contrast to LAMN emboli, which are usually found strictly adherent to them. Huang et al. studied the impact of CRS plus HIPEC followed by EPIC in LAMNs. In their study, LAMN tumors were classified as those with neoplastic epithelium present (LAMN-NEP) and those with neoplastic epithelium absent (LAMN-NEA). They found that the median survival for LAMN-NEP patients was significantly lower if they were treated with CRS plus HIPEC+EPIC compared to those treated with CRS plus HIPEC, while the median survival for LAMN-NEA patients showed a trend of better survival if they were treated with CRS plus HIPEC+EPIC, although the effect was not statistically significant [17,18,19]. Tumor biology has been extensively documented, revealing significant differences in survival rates and clinical outcomes. MUC1 and MUC2 antigens have a significant impact on a patient’s prognosis, particularly when it comes to MUC1 expression, which is often associated with poor outcomes [9].

In the majority of cases, PMP is usually asymptomatic, especially in the initial stages. When mucus builds up, it can lead to discomfort and pain in the abdomen, which may worsen with time. Regarding preoperative evaluation, numerous studies have suggested that serum tumor markers may have a predictive role. Patients with high levels of CA 19-9 are more likely to experience recurrence, and there is a clear correlation between CEA serum levels and PCI scores.

Computed tomography (CT) is the most common imaging technique in the detection of PMP. Its sensitivity depends on the tumor size and the location of tumor nodules. Although sensitivity ranges from 59 to 94%, most experts suggest that CT evaluation is the preferred imaging modality. Magnetic resonance imaging (MRI) can be used as an alternative imaging modality, but it has limitations in cases where there is involvement of the small bowel and hepatic hilar lesions [18,19,20].

There are limited data available regarding the role of PET-CT. However, PET-CT is primarily beneficial for evaluating the extent of cytoreduction and systemic metastatic disease [18].

The role of laparoscopic surgery in PMP diagnosis remains controversial. There are authors who suggest that laparoscopic evaluation is feasible and safe but also has limitations [18,19]. 

In our study, the proportion of clinically aggressive LAMN cases was higher than that reported by Mohamed et al. [9]. Recurrence developed very soon after treatment. The histopathologic characteristics of the disease remained the same, although the clinical course was particularly aggressive. Several indices, including the plasma concentration of CA-19-9 and CEA, as well as the initial PCI score, demonstrate a positive correlation with disease recurrence and overall survival (Table 2). In particular, an initially higher PCI score is a fair predictor of recurrence, while both tumor markers, CA-19-9 and CEA, are significantly associated with decreased survival. It is imperative to identify patients who present with a clinically aggressive disease to initiate more intensive treatment. This approach is crucial for achieving optimal patient outcomes and ensuring that appropriate medical care is delivered in a timely and effective manner [9,18,19,20,21]. From Table 2 we can see the differences between the group with clinically aggressive disease and the group with conventional disease. The overall survival rate of patients with mucinous peritoneal carcinomatosis does not exceed 14% [7]. In our study, three out of the four patients died within less than 2 years of their initial surgery, despite receiving systemic chemotherapy.

All previous observational reports have shown that overall survival is significantly better in patients with low-grade PMP tumors [8,21,22,23]. The series of patients in the study by Mohamed et al. included patients with DPAM tumors with an invasive clinical course who very soon relapsed despite CRS plus perioperative chemotherapy [9]. The authors investigated the correlation between tumor aggressiveness and the expression of the mucin antigens MUC1 and MUC2, but they found no difference compared to the control patients. The authors concluded that there is a subset of patients with low-grade PMP who present a clinically aggressive course, which needs further investigation at a molecular and genetic level. A prognostic gene signature for LAMNs that have metastasized to the peritoneum was identified in 2015 by Levine EA et al. This implies that such a genetic signature in the subset of more aggressive LAMNs results in significantly different clinical outcomes, even after aggressive therapy consisting of CRS and HIPEC. Pathological analysis is valuable in defining this aggressive subset, which needs to be identified. This genetic signature was found to improve patient prognosis [24].

Today, the treatment of PMP patients in specialized centers that involves CRS and perioperative intraperitoneal chemotherapy has shown that the overall survival significantly improves [8,20,21,22,23,24,25,26,27]. Centralization of PMP patients in specialized centers is required and has been suggested since 1994 by PH Sugarbaker [28,29].

### 4.1. Treatment

The goal of PMP treatment is to completely remove the visible tumor using hyperthermic intraperitoneal chemotherapy (HIPEC). Proper patient positioning is crucial to access the abdomen, preferably with the patient in the lithotomy position. A long midline incision from the xiphisternum to the pubis is usually performed, while the disease extension is assessed using the PCI. This system divides the abdomen into nine anatomical areas, with four further areas in the small bowel mesentery. A score of 0–3 is given for each of the 13 areas (0 = no tumor, 1 ≤ 0.5 cm, 2 = 0.5–5 cm, and 3 = 5 cm), and a total score (0–39) is calculated. We start with a right parietal peritonectomy with gonadal exposure and right diaphragmatic peritonectomy to mobilize the liver. This is followed by similar approaches on the left side. A radical greater omentectomy is performed inside the gastroepiploic vessels, while the spleen is carefully assessed for disease severity. A lesser omentectomy is also usually carried out. The ovaries and gallbladder are routinely removed. In some limited cases, distal gastrectomy may be performed. If achieving complete CRS is not feasible, our strategy should be to perform maximum tumor debulking (MTD) [14,15,16,17,18,19,20,21,22,23,24,25,26,27,28,29].

Completeness of cytoreduction is assessed at the end of the operating procedure using the CC score (CC0 = complete, CC1 = disease < 0.25 cm, CC2 = 0.25–2.5 cm, and CC3 ≥ 2.5 cm). Cytoreductive surgery should be discontinued when dealing with significant small bowel serosa involvement. There are several conditions that can hinder the possibility of undergoing additional surgery. These include infiltration of the pancreatic surface, ureteric obstruction, liver metastases, and the requirement for gastric resection. Another reason may be the significant involvement of the liver pedicles [1,2,3,4,5,6,7,14,15,16,17,18,19,20,21,22,23,24,25,26,27,28,29].

Once the cytoreduction procedure is finished, we proceed with intra-operative hyperthermic chemotherapy as previously mentioned in our cytoreduction protocol. Possible anastomoses are performed after the HIPEC protocol, while low rectal anastomoses are usually proceeded with an ileostomy [12,16,21,22].

### 4.2. HIPEC Regimens

Oxaliplatin is a platinum complex agent with proven toxicity in the colon and appendiceal neoplasms, and it is used in various HIPEC protocols. It appears to result in a high possibility of bleeding. In clinical practice, it is used in the Elias high-dose oxaliplatin regimen, the Glehen medium-dose oxaliplatin regimen, and the Wake Forest University oxaliplatin regimen [14,18,19]. 

Mitomycin C is an alkylating agent. It is mainly used in peritoneal malignancy, colorectal cancer, appendiceal tumors, ovarian cancer, gastric cancer, and peritoneal mesothelioma. It is currently applied in the Sugarbaker procedure, the Dutch high-dose triple dosing mitomycin C regimen, and the low-dose regimen recommended by the American Society of Peritoneal Surface Malignancies [18,19]. 

Doxorubicin is an anthracycline agent mainly used in breast cancer, bladder cancer, lymphoma, and peritoneal cancer. It is currently applied in combination with platinum agents. 

The HIPEC procedure is usually followed by perioperative chemotherapy. There are many studies that suggest combined systemic chemotherapy plus HIPEC increases the 5-year survival rate of patients with high-grade or signet-ring cell histology [18,19].

### 4.3. Follow-up

In our study, a recurring disease was observed in 25% of the patients even after the initial CC0 resection. For low-grade cases, CT scans three months after surgery and then annually were conducted for the first six years, while high-grade disease cases received more frequent screenings. Serum tumor markers are important for detecting recurrence of disease and as a prognostic tool. At present, there are no guidelines that are universally accepted about the follow-up period [9,17,18,19,20,21].

## 5. Conclusion

Pseudomyxoma peritonei (PMP) is a rare condition with a poor prognosis. Early recognition is crucial for improving oncological outcomes. The optimum treatment strategy includes cytoreductive surgery followed by a HIPEC procedure. These procedures are complex and performed only in experienced centers. If these procedures are not able to be performed, debulking surgery may be considered as an alternative option.

A small number of patients may experience an aggressive disease course, although the majority can have a long survival after receiving CRS and perioperative intraperitoneal chemotherapy. Ιdentification of these patients is challenging, but encouraging results have been shown in molecular and genetic studies. Further studies are required for the identification of this subgroup of patients.

It is important to centralize patients in dedicated centers to prevent high rates of morbidity and mortality. 

Current consensus guidelines have greatly influenced the management of PMP due to its rarity. While global recommendations may assist us in developing effective strategies, there is still a need for additional research to improve oncological outcomes. According to the 2023 PSOGI consensus in Venice, the main challenges of PMP management are achieving complete cytoreduction and managing disease recurrence.

## Figures and Tables

**Table 1:** Provides comprehensive information about the patient characteristics (study group) that were analyzed in our study.

Parameters	Patient 1	Patient 2	Patient 3	Patient 4
Gender	Male	Male	Male	Male
Age	44	40	47	63
Recurrence	Yes	Yes	No	No
Histology	LAMN	LAMN	LAMN	LAMN
ASA status	2	1	1	1
CA 19-9 IU/mL	618	581	440	460
CEA ng/mL	64	47	32	24
HIPEC (min)	90	90	No HIPEC	90
PCI	33	23	37	27
	17	6		
CC score	1	0	3	1
	3	0		
PSS score	0	0	1	1
	3	3		
Overall survival (months)	13	50 (still alive)	25	19

**Table 2:** Provide information of patient characteristics of our study and control group.

Patient Characteristics	Cases	Control	*p*-Value *
**Number of patients**	4/41	37/41	
**Gender (M/F)**	4/0	26/11	
**Median age**	48.5	49.5	0.53
**Range**	40–63	38–69	
**Smoking history**	0	7 (18.91)	
**Alcohol history**	0	0	
**Cardiovascular disease**	1 (25)	8 (21.62)	0.54
**CRS (Number)**			
**1**	2 (50)	22 (59.46)	
**2**	2 (50)	11 (29.73)	
**3**	0	4 (10.81)	
**4**	0	0	
**Mean PSS**	0.5	1.4	0.63
**Mean PCI**	30	24	0.36
**Median CA 19-9**	520.5	128	**0.041**
**Median CEA**	39.5	33.8	0.35
**Median CC Score**	1.25	0.72	0.47
**Median OS (months)**	26.75	30.63	0.74

Sign * is to emphasize the statistic significant value of 0.05.

**Table 3:** Histopatholy charecteristics of PMPs.

Lesion	Terminology
Mucin without epithelial cells	Acellular mucin
PMP with low-grade histological features	Low-grade or disseminated peritoneal adenomucinosis (DPAM)
PMP with high-grade histological features	High-grade or peritoneal mucinous carcinomatosis (PMCA)
PMP with signet-ring cells	High-grade or peritoneal mucinous carcinomatosis with signet-ring cells (PMCA-S)

## Data Availability

The datasets used and analyzed in the current study are available from the corresponding author upon reasonable request.

## Abbreviations

LAMN	Low-grade appendiceal mucinous carcinomas
CRS	Cytoreductive surgery
HIPEC	Hyperthermic intraperitoneal chemotherapy
PMP	Pseudomyxoma peritonei
DPAM	Disseminated peritoneal adenomucinosis
PMCA	Peritoneal mucinous carcinomatosis
PMCA I/D	Hybrid type of mucinous carcinomatosis
HMAC	High-grade mucinous carcinoma peritonei
PSOGI	Peritoneal Surface Oncology Group International
**CT**	Computed tomography
MRI	Magnetic resonance imaging
PET	Positron emission tomography
EPIC	Early postoperative intraperitoneal chemotherapy
